# Asthenia and coping strategies of Russian medical workers during the first wave of the novel coronavirus pandemic

**DOI:** 10.1192/j.eurpsy.2026.11011

**Published:** 2026-07-31

**Authors:** V. I. Rozhdestvenskiy, V. V. Titova, I. A. Gorkovaya, D. O. Ivanov, Y. S. Aleksandrovich

**Affiliations:** Department of Psychosomatics and Psychotherapy, Saint Petersburg State Pediatric Medical University, Saint Petersburg, Russian Federation

## Abstract

**Introduction:**

During the first wave of the COVID-19 pandemic in Russia, medical workers were forced to work much harder, quickly learn new skills, and endure constant emotional stress. These factors could have triggered the emergence of a specific psychopathological syndrome — asthenia, which is characterised by general and mental weakness, increased exhaustion, irritability, decreased mental productivity, decreased motivation, sleep disorders, and other psychosomatic disorders. To reduce asthenia, healthcare workers resorted to various coping strategies.

**Objectives:**

The study aimed to investigate the relationships between different types of asthenia and coping strategies among healthcare workers during the first wave of the COVID-19 pandemic in Russia.

**Methods:**

Data were collected between May and July 2020 using a Google Form developed by the authors. The sample comprised 45 healthcare workers residing in Russia. Levels of asthenia were assessed with the MFI-20 inventory, and coping strategies were identified using the COPE-60 inventory. Both instruments had been previously adapted for use in the Russian context.

**Results:**

We found that general asthenia had negative correlations with the coping strategies ‘behavioural avoidance’ (r_s_ = -0.407, p < 0.05) and ‘restraint’ (r_s_ = -0.396, p < 0.05); reduced activity — with the coping strategy ‘focusing on emotions and their active expression’ (r_s_ = -0.405, p < 0.05); physical asthenia — with ‘behavioural avoidance’ (r_s_ = -0.376, p < 0.05) and ‘acceptance’ (r_s_ = -0.390, p < 0.05); mental asthenia — with ‘focusing on emotions and their active expression’ (r_s_ = -0.493, p < 0.01) and ‘use of “sedatives”’ (r_s_ = -0.478, p < 0.01). Decreased motivation had no statistically significant correlations with any coping strategy.

**Conclusions:**

During the first wave of the pandemic, Russian medical professionals exhibited the following pattern. As they increasingly abandoned active attempts to change the situation and adopted a wait-and-see attitude, as well as accepting what had happened rather than repressing it, their levels of fatigue and physical asthenia decreased. The pace of work among medical workers was negatively associated with the degree to which they demonstrated their emotions vividly. As mental asthenia increased, medical workers showed their emotions less vividly and resorted to psychoactive substances less often. It is necessary to train medical workers in practical ways to combat asthenia.

**Disclosure of Interest:**

None Declared

